# Relationship Between Glycated Hemoglobin (HbA1c) and Vitamin D Levels in Type 2 Diabetes Patients: A Retrospective Cross-Sectional Study

**DOI:** 10.7759/cureus.62468

**Published:** 2024-06-16

**Authors:** Basheer Abdo, Mohammed Abdullah, Ismaeel A AlShoaibi, Faisal Ahmed, Redwan Alawdi, Khaled Alzanen, Kamal Algaadi

**Affiliations:** 1 Department of Internal Medicine, School of Medicine, Ibb University, Ibb, YEM; 2 Department of Urology, Ibb University, Ibb, YEM

**Keywords:** glycated hemoglobin, deficiency, diabetes mellitus, yemen, ibb city, vitamin d

## Abstract

Background: Significant links between low serum levels of vitamin D3 and insufficient glycemic control in patients with type 2 diabetes mellitus (T2DM) have been reported previously in the literature. However, there is no exciting evidence on the association between glycated hemoglobin (HbA1c) and vitamin D levels in T2DM individuals in our nation (Yemen). This study aimed to investigate the relationship between HbA1c and vitamin D levels in T2DM patients in a resource-limited setting.

Method: A retrospective cross-sectional study was conducted at the Al-Raffa Center, Ibb, Yemen between June 2018 and September 2023 including 396 patients diagnosed with T2DM. The patient characteristics, comorbidities, HbA1c, and vitamin D levels were gathered from patients' medical profiles. Linear regression analysis was used to find the factors associated with vitamin D deficiency (serum 25(OH)D levels < 20 ng/mL) among T2DM patients. Subsequently, the correlation between HbA1c and vitamin D levels was examined using receiver operating characteristic (ROC) curve analysis.

Results: The mean age was 44.6 ±14.6 years and most of them (n= 227, 57.3%) were female and from a rural area (n= 229, 57.8%). Comorbidities were hypertension, dyslipidemia disease, and cardiovascular disease in 176 (44.4%), 63 (15.9%), and 88 (22.2%) cases, respectively. The mean HbA1c was 8.1 ±2.5%. The mean vitamin D level was 26.9 ±16.5 ng/mL and low vitamin D was present in 260 (65.7%) (vitamin D deficiency in 160 (40.4%) and vitamin D insufficiency in 100 (25.3%) cases). In regression analysis, obesity (>30 kg/m^2^) (odds ratio (OR): 299.49; 95% confidence interval (CI): 72.66 - 1234.42, p <0.0001), higher HbA1c levels (OR: 1.61; 95% CI: 1.26 - 2.05, p =0.0001), and urban residence (OR: 23.98; 95% CI: 5.62 - 102.42, p <0.0001) were associated with vitamin D deficiency. There was a negative correlation between the vitamin D level and HbA1c which was statistically significant (correlation coefficient r: -0.5452; 95% CI: -0.6109 to -0.4720, p <0.0001). Using the ROC analysis, the serum vitamin D value of ≤18.42 ng/ml was the best cut-off point to predict hyperglycemia (area under the curve: 0.633, 95% CI: 0.672 to 0.770, sensitivity: 52%, specificity: 84.71 %, Yoden’s index: 0.3671, p <0.001). Based on this cut-off, 39.4% of individuals (37.5% in the normoglycemic group and 90.9% in the hyperglycemic group) were vitamin D deficient.

Conclusion: In this study, low vitamin D was common among T2DM patients, especially those with poor glycemic control. We observed a link between HbA1c levels, urban residency, and BMI with vitamin D deficiency in T2DM patients. The association was distinguished by low vitamin D levels and elevated HbA1c. Additionally, we found that the serum vitamin D value of ≤18.42 ng/ml was the best cut-off point to predict hyperglycemia in T2DM patients with moderate agreement. To manage their disease, patients with T2DM should take their medications as prescribed and live a healthy lifestyle. This will increase their overall health, especially their vitamin D levels.

## Introduction

Vitamin D, a fat-soluble vitamin essential for human biological activities, is produced in animals, fungi, and plant tissues when exposed to sunlight. Vitamin D is classified as a steroid and a precursor molecule, promoting the production of human steroid hormones [1]. Diet and sun exposure are the primary sources of vitamin D [2,3]. Ergocalciferol (D2) is present in vitamin D-rich foods such as cod liver oil, whereas cholecalciferol (D3) is created by dermal synthesis. The liver enzyme 25-hydroxylase transforms D2 and D3 into active vitamin D, whereas the kidney enzyme 25-hydroxyvitamin D-1 alpha-hydroxylase facilitates the process [3]. Vitamin D is essential for musculoskeletal growth and function, as it directly influences phosphorus and calcium absorption [3]. Vitamin D receptors are located in many organs, including the digestive, immunological, neurological, and musculoskeletal systems. These receptors activate or deactivate certain genes, promoting phosphorus and calcium absorption in the gastrointestinal system [4]. Additionally, vitamin D is essential for modulating immune system abnormalities such as responses to inflammation and autoimmune disorders, as well as downregulating adaptive immunological processes. It improves immunological tolerance and affects glucose metabolism and homeostasis, including insulin production [5].

Previous studies have found significant links between insufficient glycemic management and various issues in patients with type 2 diabetes mellitus (T2DM) implying that greater levels of vitamin D levels lead to improved glucose homeostasis management [6-10]. Alotaibi et al. found a significant association between vitamin D deficiency and higher glycated hemoglobin (HbA1c) and hyperlipidemia in T2DM patients [2]. A recent umbrella meta-analysis found that vitamin D supplementation significantly reduced fasting blood sugar, HbA1c, insulin concentrations, and homeostatic model assessment for insulin resistance and suggested that vitamin D supplementation may be beneficial for diabetes mellitus (DM) management [11].

Diabetes is regarded as a major public health concern around the world, including our country. In Yemen, which has a population of approximately 23 million, the prevalence of diabetes was estimated to be 9.75% among people aged ≥20 years in 2014 [12]. However, there is no exciting evidence on the association between HbA1c and vitamin D levels in T2DM individuals in our nation. This study aimed to investigate the relationship between HbA1c and vitamin D levels in T2DM patients in a resource-limited setting.

## Materials and methods

Study design and setting

A retrospective cross-sectional study was conducted at the Al-Raffa Center, Ibb, Yemen between June 2018 and September 2023 including patients diagnosed with T2DM who were evaluated for HbA1c, and vitamin D levels were gathered and analyzed from medical profiles. 

Inclusion criteria

Adult T2DM patients, based on the American Diabetes Association's (2014) diagnostic criteria [13], aged ≥18 years who presented at the Al-Raffa Center, Ibb, Yemen during the study period were included in this study.

Exclusion criteria

Pregnant women, patients with metabolic syndrome, and malabsorptive statuses were excluded.

Sample size

The sample size was calculated using G Power version 3 software and selected by setting a 95% confidence interval (CI), 80% power, 76.6% expected frequency of vitamin D deficiency in cases of T2DM, and 5% alpha error and based on a previously reported by Alhumaidi et al. [14]. The sample size was determined to be 266 patients. Given the drop rate of 30% for the expected non-response, the total calculated sample size was 396 participants.

Collected data and definitions

The collected data include age, gender, residency, educational level, body mass index (BMI), comorbidities (hypertension, cardiovascular diseases (CVDs), dyslipidemia), and laboratory results of HbA1c and vitamin D levels. HbA1c categories for controlled (less than 5.7%), prediabetic status (between 5.7% and 6.4%), and diabetes Mellitus (greater than 6.4%). The BMI categories for normal, overweight, and obesity were 18-24.9 kg/m^2^, 25-29.9 kg/m^2^, and ≥ 30 kg/m^2^. Vitamin D deficiency was defined as serum 25(OH)D levels < 20 ng/mL, whereas vitamin D more than 20 ng/mL was defined as the normal value [8].

Main outcome

The main outcome was to find the factors associated with vitamin D deficiency (serum 25(OH)D levels < 20 ng/mL) among T2DM patients. The secondary outcome was to investigate the correlation between vitamin D and HbA1c levels.

Statistical analysis

For quantitative variables, we employed the mean± SD for descriptive purposes, while qualitative variables were characterized by their frequencies. To verify the normal distribution of the study's variables, we utilized the Kolmogorov-Smirnov test. To discern significant associations between qualitative variables, we implemented the chi-square test. In instances where the expected frequency was restricted, Fisher's exact test was deemed appropriate. Also, a binary logistic regression was used for variables predicting vitamin D deficiency and showing their relationship with each other. The odds ratio (OR) and 95% CI were used to show the effect size in the model. Pearson's correlation coefficient was used to determine the relationship between vitamin D levels and HbA1c levels. A receiver operating characteristic (ROC) curve analysis was utilized to determine the appropriate serum vitamin D value in T2DM with the highest sensitivity and specificity. The optimal cutoff values were the serum vitamin D concentration at the related criterion of the highest Youden Index from the ROC analysis using MedCalc Software Ltd (MedCalc Software bvba, Ostend, Belgium). All analyses were done using IBM SPSS Statistics for Windows, Version 23 (Released 2015; IBM Corp., Armonk, New York, United States) and the significance level was also considered (p < 0.05).

Ethical approval

The study was adherent to the Helsinki Declaration's principles, and Ibb University's ethics board approved this research (Code: IBBUNI. AC. YEM. 2024.71 on 2024-02-03). Patients were briefed about the research objectives, and written consent was obtained before data capturing.

## Results

The mean age was 44.6 ±14.6 years (range 18.0 - 88.0 years) and most of them (n= 227, 57.3%) were female and from a rural area (n= 229, 57.8%). The educational levels were illiterate, middle school or below, and high school or college in 71 (17.9%), 188 (47.5%), and 137 (34.6%) cases, respectively. The mean HbA1c was 8.1 ±2.5% (range 4.0 - 14.1%). The mean vitamin D level was 26.9 ±16.5 ng/mL and low vitamin D was present in 260 (65.7%) (vitamin D deficiency in 160 (40.4%) and vitamin D insufficiency in 100 (25.3%) cases) (Table 1).

**Table 1:** Baseline and laboratory variables of diabetic patients (n=396). HbA1c: glycated hemoglobin, SD: standard deviation, IQR: interquartile range, n: number.

Variable	N (%)
Age (year), Mean ±SD	44.6±14.6
Age (year), Median (IQR)	43 (33, 55)
Gender	
Female	227 (57.3%)
Male	169 (42.7%)
Educational level	
Illiterate	71 (17.9%)
Middle school or below	188 (47.5%)
High school or college	137 (34.6%)
Residence	
Rural	229 (57.8%)
Urban	167 (42.2%)
Body mass index	
Normal weight (18-24.9 kg/m^2^)	138 (34.8%)
Overweight (25 to 29.9 kg/m^2^)	160 (40.4%)
Obesity (>30 kg/m^2^)	98 (24.7%)
Comorbidities	
Hypertension	176 (44.4%)
Cardiovascular disease	63 (15.9%)
Dyslipidemia disease	88 (22.2%)
HbA1c (%), Mean ±SD	8.1 ±2.5
Vitamin D levels (ng/mL), Mean ±SD	26.9 ±16.5
Normal Vitamin D	136 (34.3%)
Low Vitamin D	260 (65.7%)
Vitamin D deficiency	160 (40.4%)
Vitamin D Insufficiency	100 (25.3%)

Factors associated with vitamin D deficiency

In univariate analysis, Pearson's Chi-squared test showed that urban residency (p< 0.001), body mass index (p< 0.001), dyslipidemia disease (p= 0.036), and HbA1c (p< 0.001) were associated with vitamin D deficiency (Table 2).

**Table 2:** Baseline and laboratory variables of diabetic patients with and without vitamin D deficiency in univariate analysis. Note: Data presented as means or proportions with percentages and 95% confidence intervals in brackets. The differences between groups were analyzed using ANOVA^1^ and Pearson's Chi-square test^2^ as appropriate. Bold values indicate statistically significant results (p < 0.05). SD: standard deviation, HbA1c: glycosylated hemoglobin, OR: odds ratio, CI: confidence interval.

Variable	Subgroup	Non-vitamin D deficient (N=136)	Vitamin D deficient (N=260)	OR (95%CI)	p-value
Age (year)	Mean ± SD	46.2 ±14.3	43.7 ±14.6	0.99 (0.97-1.00)	0.107^1^
Gender	Male	79 (58.1)	148 (56.9)	Reference group	0.824^2^
	Female	57 (41.9)	112 (43.1)	1.05 (0.69-1.60)	
Educational level	Middle school or below	72 (52.9)	116 (44.6)	Reference group	0.128^2^
	Illiterate	26 (19.1)	45 (17.3)	1.07 (0.61-1.91)	
	High school or college	38 (27.9)	99 (38.1)	1.62 (1.01-2.62)	
Residence	Rural	122 (89.7)	107 (41.2)	Reference group	<0.001^2^
	Urban	14 (10.3)	153 (58.8)	12.46 (7.01-23.73)	
Body mass index	Normal weight (18-24.9 kg/m^2^)	129 (94.9)	9 (3.5)	Reference group	<0.001^2^
	Overweight (25 to 29.9 kg/m^2^)	5 (3.7)	155 (59.6)	444.33 (159.23-1528.7)	
	Obesity (>30 kg/m^2^)	2 (1.5)	96 (36.9)	688.00 (179.85-4631.00)	
Cardiovascular disease	No	116 (85.3)	217 (83.5)	Reference group	0.742^2^
	Yes	20 (14.7)	43 (16.5)	1.15 (0.65-2.08)	
Hypertension	No	71 (52.2)	124 (47.7)	Reference group	0.455^2^
	Yes	65 (47.8)	136 (52.3)	1.20 (0.79-1.82)	
Dyslipidemia disease	No	114 (83.8)	194 (74.6)	Reference group	0.036^2^
	Yes	22 (16.2)	66 (25.4)	1.76 (1.05-3.06)	
HBA1C (%)	Mean ±SD	6.6 ±1.7	8.9 ±2.5	1.59 (1.42-1.80)	<0.001^1^

In linear regression analysis, obesity (>30 kg/m^2^) (OR: 299.49; 95% CI: 72.66 - 1234.42, p <0.0001), higher HBA1C levels (OR: 1.61; 95% CI: 1.26 - 2.05, p =0.0001), and urban residence (OR: 23.98; 95% CI: 5.62 - 102.42, p <0.0001) were associated with low vitamin D (Table 3).

**Table 3:** Characteristics of patients with and without vitamin D deficiency in liner regression analysis (n=396). Notes: Data are expressed as a linear regression coefficient (95% confidence interval). Bold values indicate statistically significant results (p < 0.05). HbA1c: glycosylated hemoglobin, OR: odds ratio, CI: confidence interval.

Variable	Coefficient	Std. Error	Wald	OR	95% CI	P-value
Body mass index	5.70211	0.72258	62.2731	299.49	72.66 to 1234.42	<0.0001
Residence	3.17744	0.74065	18.4045	23.98	5.62 to 102.42	<0.0001
HbA1c	0.47720	0.12348	14.9351	1.61	1.26 to 2.05	0.0001
Dyslipidemia	-0.31477	0.73519	0.1833	0.73	0.17 to 3.08	0.6685

Correlation between the vitamin D level and HbA1c levels

**Figure 1:**
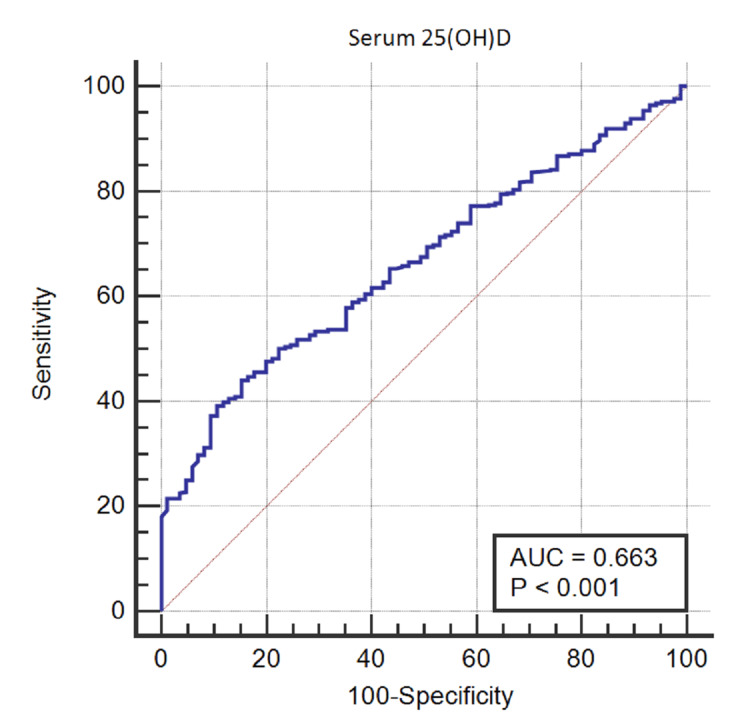
The results of receiver operating characteristic curve analysis of the serum vitamin D threshold levels. AUC: Area under the curve

There was a negative correlation between the vitamin D level and HbA1c and it was statistically significant (Correlation coefficient r: -0.5452; 95% CI: -0.6109 to -0.4720, p <0.0001) which suggests that increased vitamin D levels were connected with lower HbA1c levels. Using the ROC analysis for the entire group, the serum vitamin D value of ≤18.42 ng/ml was the best cut-off point to predict hyperglycemia in the T2DM patients with moderate agreements (area under the ROC curve (AUC): 0.633, 95% CI: 0.672 to 0.770, sensitivity: 52%, specificity: 84.71 %, Yoden’s index: 0.3671, p <0.001). Based on this cut-off, 39.4% of individuals (37.5% in the normoglycemic group and 90.9% in the hyperglycemic group) were vitamin D deficient (Figure 1).

## Discussion

In this study, we retrospectively looked at the relationship between deficiencies in vitamin D and HbA1c, as well as the relationship between vitamin D deficiency and other variables in T2DM patients in a resource-limited situation. Our result showed that 21.5% of patients had normal HbA1c values, suggesting good glycemic control, while 63.1% had considerably higher HbA1c levels, indicating poor glycemic control. Noteworthily, previous studies have linked suboptimal glycemic control to factors like older age, vitamin D levels, illiteracy, comorbidities, insufficient self-monitoring of blood glucose levels, insufficient physical activity, hyperlipidemia, antidiabetic drugs, longer diabetes duration, and elevated waist-to-hip ratio [15,16]. Our findings were consistent with previous local and Middle East studies that indicated poor glycemic control among individuals with T2DM based on HbA1c levels [10,16,17]. These findings highlight the severe condition of inadequate glycemic control in our patients, which requires attention due to the negative health consequences. However, due to the study's retrospective monocentric design and small sample size, the results should be interpreted with caution.

We found that lower levels of serum 25(OH) vitamin D were found to be associated with higher levels of HbA1c (Correlation coefficient r: -0.5452; 95% CI: -0.6109 to -0.4720, p <0.0001). This study's negative link between vitamin D and HbA1c levels lends support to earlier reports that have established an association between T2DM and vitamin D status [6-10,18]. This negative correlation might be due to vitamin D deficiency causing glucose intolerance and resistance to exogenous insulin.

Vitamin D deficiency and insufficiency were defined by the Endocrine Society as serum 25(OH)D values below 20 ng/ml and 21-29 ng/ml, respectively. A level of at least 30 ng/ml was considered sufficient [19]. However, guidelines for interpreting vitamin D levels must consider non-bone-related outcomes like hypertension, BMI, cardiovascular diseases (CVDs), and glucose and insulin metabolism measurements [15,16]. A prior study established a criterion for low vitamin D based on fasting blood glucose levels. Alaei-Shahmiri et al. discovered that a serum 25(OH)D level of approximately 15 ng/ml was the ideal cut-off point for predicting hyperglycemia among the Iranian population [20]. In our study, using the ROC analysis, the serum vitamin D value of ≤18.42 ng/ml was the best cut-off point to predict hyperglycemia in the T2DM patients with moderate agreements (AUC: 0.663, 95% CI: 0.672 to 0.770, sensitivity: 52%, specificity: 84.71 %, Yoden’s index: 0.3671, p <0.001). Based on this threshold, 39.4% of individuals including 37.5% and 90.9% in normoglycemic and hyperglycemic groups were vitamin D deficient respectively. However, this cut-off point was higher than the threshold of 15 ng/ml suggested by Alaei-Shahmiri et al. to predict hyperglycemia among the Iranian population [20]. In another study, Ren et al. reported an ideal cut-off value for serum 25-OHD of 23.14 ng/ml with a sensitivity, specificity, and AUC of 64.71%, 70.59%, and 0.721, respectively among patients with gestational DM [21]. The findings from these reports recommended that individualized vitamin D supplementation could be an effective future therapy for preventing poor glucose control [22].

In the present study, there was no correlation between vitamin D deficiency and age. Our findings were consistent with those of Salih et al. who mentioned no significant relationship between age and vitamin D levels [23]. In contrast, Enverga et al. found a significant but slight positive correlation between serum vitamin D levels and patient age. Their HbA1c analysis revealed that those with high HbA1c and low vitamin D were younger, even after controlling factors that influenced vitamin D levels in the study sample [18]. This discrepancy may be attributed to the socioeconomic conditions specific to our regions, as most cases were young (median age of 43 years). Advanced age affects vitamin D and calcium metabolism by causing calcium malabsorption, intestinal resistance, diminished vitamin D receptors, impaired renal synthesis, and decreased vitamin D skin production [11].

We found no statistically significant correlations between gender, level of education, and comorbidities like hypertension, CVDs, and dyslipidemia with vitamin D deficiency. However, some of these factors were reported to be associated with vitamin D deficiency with controversies in different studies [8,18,23-26]. As a result, it is critical to understand that variances in reported results may be due to a lack of standardization, methodological differences, or research population heterogeneity. More studies with larger sample sizes and including more variables are required to clarify these concerns. BMI, particularly obesity, was found as a risk factor for low serum vitamin D levels [11].

In this study, obesity (>30 kg/m^2^) was associated with vitamin D deficiency (OR: 299.49) and was statistically significant. A similar result from Middle Eastern studies supports this finding. For example, Alloubani et al. found a link between high BMI, low vitamin D levels, and diabetes [27]. The justification could be owing to adipose tissue's high tendency to sequester vitamin D, making it biologically inaccessible. Another probable explanation is leptin levels in the serum. In vivo experiments show that leptin reduces 1α(OH)ase and 24(OH)ase activity while correcting increased calcium, phosphate, and 1,25(OH)2D3 levels. This suggests that leptin has a role in lowering 1,25(OH)2D3 synthesis. Surprisingly, the blood leptin level is favorably linked with obesity. As a result, in the presence of obesity, 1,25(OH) 2D3 levels may be excessively down-regulated, potentially leading to vitamin D deficiency [28,29]. In contrast, another study revealed no significant link between BMI categories and vitamin D levels [26]. Future research is needed to accurately assess the relationship between BMI and vitamin D levels.

Vitamin D deficiency may be an additional health disparity encountered by both rural and inner-city veterans, and patients living in both areas should be regarded at high risk for vitamin D deficiency and evaluated regularly [30]. In this study, urban residences were statistically significant and associated with vitamin D deficiency (OR: 23.98). A similar result was mentioned by Maddah et al., who mentioned that vitamin D inadequacy was more common in urban areas, especially in the upper social class [31]. The urban-rural vitamin D status gap is attributable to differences in living conditions and housing, with urban staying indoors and rural working as farmers, resulting in increased personal solar exposure than urban individuals. Food fortification, dietary supplements, and sun exposure are all necessary for increasing vitamin D levels, but not all are accessible or used in our city due to poverty and bad social habits such as khat chewing. Yemeni women, on the other hand, are rarely exposed to the sun due to their wardrobe choices. It has been proposed that the entire body covered with voluminous clothes among Muslim women and adolescent girls may be linked to the high frequency of vitamin D deficiency in Islamic nations [31,32].

There are additional factors that are predictors of vitamin D deficiency among patients with T2DM. For example, in a recent systematic review and meta-analysis, Taderegew et al. mentioned that diabetic-related microvascular complications such as albuminuria, nephropathy, and retinopathy were predictors of vitamin D deficiency among patients with T2DM [29]. Unfortunately, some of these factors were not collected during the study period. For that, we recommended future prospective studies include these factors and investigate their effects on vitamin D deficiency among patients with T2DM.

Study limitations

The current study had several limitations. The reliance on secondary data, the quality of which can be inconsistent due to variations in documentation, data integrity, and record-keeping practices are the main boundaries. Furthermore, the retrospective nature of the study may introduce inherent biases. Excluding records with incomplete data could also potentially introduce selection bias into the analysis. Additionally, disparate geographical populations and cultural contexts may contribute to the divergent factors influencing induction outcomes. To mitigate these limitations and provide more robust findings, we recommend conducting a prospective study with a larger sample size and extended post-treatment follow-up. Nevertheless, this study is the first to report on vitamin D deficiency in T2DM patients in Yemen, providing a reliable overview of prevalence and associated factors. Further research is needed to understand the specific needs and prospects of vitamin D deficiency among T2DM patients in resource-limited settings.

## Conclusions

In this study, low vitamin D was common among T2DM patients, especially those with poor glycemic control. We observed a link between HbA1c levels, urban residency, and BMI with vitamin D deficiency in T2DM patients. The association was distinguished by low vitamin D levels and elevated HbA1c. Additionally, we found that the serum vitamin D value of ≤18.42 ng/ml was the best cut-off point to predict hyperglycemia in T2DM patients with moderate agreement. To manage their disease, patients with T2DM should take their medications as prescribed and live a healthy lifestyle. This will increase their overall health, especially their vitamin D levels.
